# A method to determine and simulate the permeation through a gel matrix in a multi-organ-chip

**DOI:** 10.1186/1753-6561-9-S9-P77

**Published:** 2015-12-14

**Authors:** Hao-Hsiang Hsu, Laura E Harder, Gerd Lindner, Uwe Marx, Ralf Pörtner

**Affiliations:** 1 Hamburg University of Technology, Institute of Bioprocess- and Biosystems Engineering, Germany; 2 Technische Universität Berlin, Institute of Biotechnology, Germany

## Background

The Multi-Organ-Chip (MOC) is a new micro size bioreactor. It offers two tissue cultivation segments (e.g. skin model, liver cells), which are connected by a dynamic micro pump system. [1,2]

One future application of this system is the dermal application of substances for drug testing in vitro. In this study a method to simulate and validate the permeation of fluorescein sodium salt through a barrier in the MOC was established. Fluorescein sodium salt has a molecular weight of 376.28 g/mol which is in the range of steroid hormones like testosterone (288.4 g/mol), aldesterone (360.4 g/mol), hydrocortisone (362.5.4 g/mol) and corticosterone (346 g/mol) [3]. Furthermore, first permeation experiments through four cell-collagen models, as reference for skin models, were carried out.

## Methods

**Figure 1:**
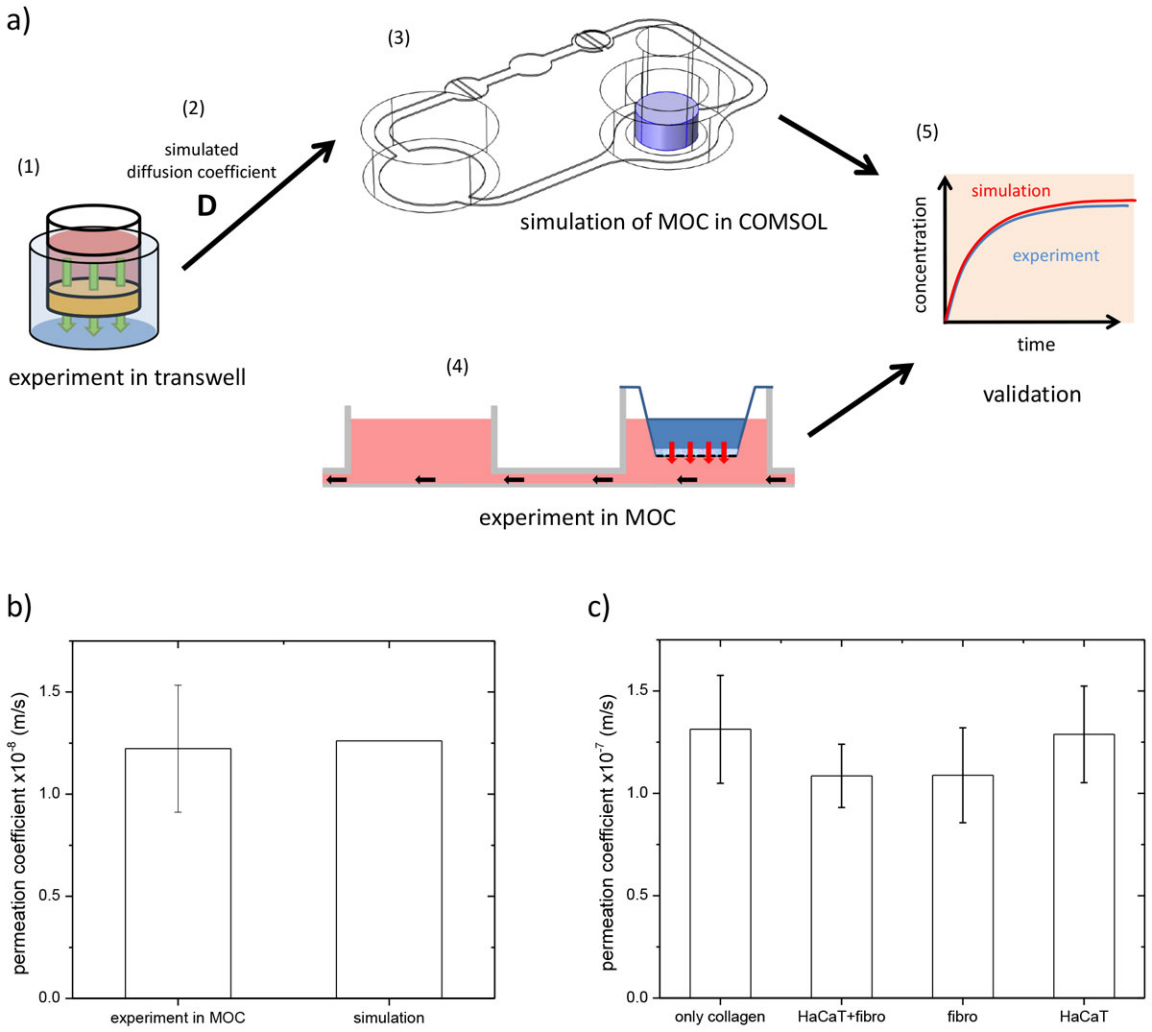
**a) Schematic figure of the method**. b) Results of the validation: experiment and simulation were done with 2% agarose gel and fluorescein sodium salt. c) Results of permeation experiment in four different collagen - cell models with fluorescein sodium salt.(n = 2x3)

Fig. 1 a) shows the method of the permeation experiment, simulation and validation. The detail will be explained as follow step by step: (1) Permeation experiments were performed in a 12 transwell system with 2 % agarose gel as a skin model substitute. Fluorescein sodium salt was applied on top of the gel as it can be detected by fluorescence measurements and hence, sampling is not necessary. The concentration in the acceptor below the gel can be detected by fluorescence measurements. The experiment is carried out in an incubator at 37 °C on a shaker. (2) The simulation program COMSOL Multiphysics was used to determine the diffusion coefficient of the permeation experiment. (3) Subsequently the MOC was integrated in the simulation. The diffusion coefficient of the previous calculation was set as constant. (4) Additionally to the simulation, a permeation experiment was executed in the MOC with the same setting as the experiment in the 12 transwells. (5) The simulation was validated by comparing simulated vs experimental data. (see Fig. 1 a))

To test the feasibility of the permeation experiment with skin models, four different setups with a HaCaT cell line and primary fibroblasts (HaCaT+fibro, fibro, HaCaT and only collagen) were established. Therefore, the cells were immobilized in collagen gel. The experiment was also performed in a 12 transwell system using fluorescein sodium salt.

## Results

The standard deviation of the experiment with the MOC is high with approximately 25 %. Possible reason could be attributed to the experiment a setting by slight variation of the gel and positioning of the transwell in the MOC.

Fig. 1 b) shows the results of the simulation validation with fluorescein sodium salt. There is a accordance indicated by the mean permeation coefficient of the experiments and the simulation with a standard deviation of 3.05 %. The results of the experiment with cell-collagen setups is shown on Fig.1 c). The permeation coefficients are 1.31 ± 0.26 x10-7 m/s for only collagen, 1.09 ± 0.15 x10-7 m/s for HaCaT+Fibro, 1.09 ± 0.23 x10-7 m/s for Fibro and 1.29 ± 0.24 x10-7 m/s for HaCaT. There is no significant difference between the setups but a trend can be seen. The gels with fibroblasts seem to have a lower permeation property. In comparison to agarose gel the permeation coefficient in cell-collagen model is ten times lower (data not shown).

## Outlook

A method for the simulation of diffusion processes in a MOC with permeation experiment was established. First validations show a good fitting of the simulation. Further validation experiments with different substances should be done to determine the range of application of the simulation.

A reproducible method in transwell systems was established. Further permeation experiments and simulations will be carried out with skin models.

